# Regulation of lipolysis by 14-3-3 proteins on human adipocyte lipid droplets

**DOI:** 10.1093/pnasnexus/pgad420

**Published:** 2023-12-06

**Authors:** Qin Yang, Zinger Yang Loureiro, Anand Desai, Tiffany DeSouza, Kaida Li, Hui Wang, Sarah M Nicoloro, Javier Solivan-Rivera, Silvia Corvera

**Affiliations:** Program in Molecular Medicine, University of Massachusetts Chan Medical School, Worcester, MA 01605, USA; Morningside Graduate School of Biomedical Sciences, University of Massachusetts Chan Medical School, Worcester MA 01605, USA; Program in Molecular Medicine, University of Massachusetts Chan Medical School, Worcester, MA 01605, USA; Morningside Graduate School of Biomedical Sciences, University of Massachusetts Chan Medical School, Worcester MA 01605, USA; Program in Molecular Medicine, University of Massachusetts Chan Medical School, Worcester, MA 01605, USA; Program in Molecular Medicine, University of Massachusetts Chan Medical School, Worcester, MA 01605, USA; Program in Molecular Medicine, University of Massachusetts Chan Medical School, Worcester, MA 01605, USA; Program in Molecular Medicine, University of Massachusetts Chan Medical School, Worcester, MA 01605, USA; Program in Molecular Medicine, University of Massachusetts Chan Medical School, Worcester, MA 01605, USA; Program in Molecular Medicine, University of Massachusetts Chan Medical School, Worcester, MA 01605, USA; Program in Molecular Medicine, University of Massachusetts Chan Medical School, Worcester, MA 01605, USA; Diabetes Center of Excellence, University of Massachusetts Chan Medical School, Worcester, MA 01605, USA

**Keywords:** lipid droplet, proximity labeling, 14-3-3 proteins, lipolysis, insulin

## Abstract

Adipocyte lipid droplets (LDs) play a crucial role in systemic lipid metabolism by storing and releasing lipids to meet the organism's energy needs. Hormonal signals such as catecholamines and insulin act on adipocyte LDs, and impaired responsiveness to these signals can lead to uncontrolled lipolysis, lipotoxicity, and metabolic disease. To investigate the mechanisms that control LD function in human adipocytes, we applied proximity labeling mediated by enhanced ascorbate peroxidase (APEX2) to identify the interactome of PLIN1 in adipocytes differentiated from human mesenchymal progenitor cells. We identified 70 proteins that interact specifically with PLIN1, including PNPLA2 and LIPE, which are the primary effectors of regulated triglyceride hydrolysis, and 4 members of the 14-3-3 protein family (YWHAB, YWHAE, YWHAZ, and YWHAG), which are known to regulate diverse signaling pathways. Functional studies showed that YWHAB is required for maximum cyclic adenosine monophosphate (cAMP)-stimulated lipolysis, as its CRISPR-Cas9-mediated knockout mitigates lipolysis through a mechanism independent of insulin signaling. These findings reveal a new regulatory mechanism operating in human adipocytes that can impact lipolysis and potentially systemic metabolism.

**Significance Statement**

Lipid droplets (LDs) are ubiquitous cytoplasmic organelles that store metabolic energy and play a key role in cellular lipid metabolism (1). Adipocyte LDs play an additional, crucial role, as they supply the energy needs of the whole body through hormonally regulated triglyceride synthesis, storage, and release. The mechanisms by which adipocyte LDs release lipids for systemic use have been mostly studied in mouse models and cell lines. To understand how lipid mobilization is controlled in human adipocytes, we used proximity labeling to identify proteins that interact with PLIN1, a major component of the LD, in adipocytes generated from primary human progenitor cells. Our study catalogs the interactome of human PLIN1 and identifies a previously unrecognized potential mechanism for control of human adipocyte lipolysis through a specific 14-3-3 protein, YWHAB.

## Introduction

Lipid droplets (LDs) are unique organelles that store neutral lipids, have a ubiquitous presence in eukaryotic cells, and are considered central hubs for cellular lipid metabolism, signal transduction and trafficking events (1–3). In adipocytes, LDs play the additional key role of providing for the storage, and supply needs of the whole organism, a function that is controlled largely through catecholamine and insulin signaling. Under fed conditions, insulin inhibits lipolysis and promotes esterification of fatty acids into triglycerides, which are stored in the large LDs characteristic of differentiated adipocytes. The failure to store lipids appropriately in adipocytes can lead to excessive lipid accumulation in cells of other tissues such as liver, muscle, and heart, leading to lipotoxic oxidative stress and mitochondrial damage during autophagy (4). Under conditions of starvation, exercise, or cold exposure, neutral lipids stored in LDs are rapidly mobilized and enter the circulation to provide for the energy needs of other tissues. Given the central role of adipocyte LDs in systemic energy homeostasis, dysregulation or dysfunction of LDs has a major impact on human metabolic diseases such as obesity, diabetes, cardiovascular disease, and nonalcoholic fatty liver disease.

In mammalian cells, LDs consist of a hydrophobic core of neutral lipids, such as sterol esters and triacylglycerols, surrounded by a phospholipid monolayer decorated by numerous specific proteins (2, 3). During feeding conditions, fatty acids are esterified into triglycerides in the endoplasmic reticulum and directed to LDs for storage by mechanisms that are controlled by insulin. Under fasting, exercise, or cold exposure, catecholamine signaling stimulates the hydrolysis of LD triglycerides into fatty acids and glycerol (5) in a process mediated sequentially by the three lipolytic enzymes adipose triglyceride lipase (ATGL, *PNPLA2*) (6), hormone-sensitive lipase (HSL, *LIPE*) (7), and monoacylglycerol lipase (MGL, *MGLL*) (7). The regulation of fatty acid esterification and triglyceride lipolysis is mediated by proteins that interact with the surface of the LD. Under basal conditions, perilipin 1 (*PLIN1*), the most abundant protein localized on the surface of LDs (8–10), sequesters CGI-58, a coactivator of ATGL, preventing lipase activity (11). In response to catecholamines, PLIN1 becomes phosphorylated by protein kinase A (PKA), releasing CGI-58 which can then activate ATGL to initiate lipolysis (11). HSL is also phosphorylated by PKA and then translocated from the cytosol to the surface of LDs, thereby exerting the hydrolytic enzyme activity.

This most widely accepted model of lipolysis regulation is mainly derived from murine cells and mouse genetic models, but whether identical mechanisms operate in human adipocytes is largely unknown. There are important differences between mouse and human adipocytes, including significant differences in cell and LD size which could influence regulatory mechanisms. Indeed, in a previous study, we found that a primate-specific long noncoding RNA, *LINC00473* colocalizes with PLIN1 and positively regulates stimulated lipolysis (12), but an analogous molecule has not been identified in mice. A detailed understanding of LD proteome dynamics triggered by lipolytic stimuli can ultimately help us to reveal the human-specific mechanisms controlling lipid mobilization in a more comprehensive way.

The identification of LD proteins has predominantly been accomplished using subcellular fractionation combined with proteomic analyses, using mouse clonal cell lines (13, 14) or mouse adipose tissues (15, 16). More recently, proximity labeling by APEX2 (17, 18) has been developed as a powerful technique to identify the interactome of specific proteins in intact cells. Using H_2_O_2_ as a cosubstrate, APEX2 rapidly oxidizes biotin–phenol (BP) into a highly reactive biotin–phenoxyl radical, which diffuses outward to covalently tag proximal endogenous proteins within 10 nm with fast kinetics (<1 min) and high activity. PLIN2-APEX2 and ATGL(S47A)-APEX2 chimeras were used recently to characterize the composition of the LD proteome in two different cell lines, U2OS and Huh7 (19). However, these cells do not exhibit the large, hormonally responsive LD characteristics of adipocytes. Here, we used a similar approach to identify the PLIN1 interactome in human adipocyte LDs under different conditions of hormonal stimulation. We leveraged techniques to obtain human adipose tissue-derived progenitor cells at scale (18), which have the capacity to differentiate into at least four adipocyte subtypes (20). Our studies identify additional members of the LD proteome, including four members of the 14-3-3 family (YWHAB, YWHAE, YWHAG, and YWAHZ). Functional studies reveal that YWHAB modulates cyclic adenosine monophosphate (cAMP)-induced stimulated lipolysis and the antilipolytic action of insulin.

## Results

### Generation and characterization of APEX2 fusion constructs

**Fig. 1.**
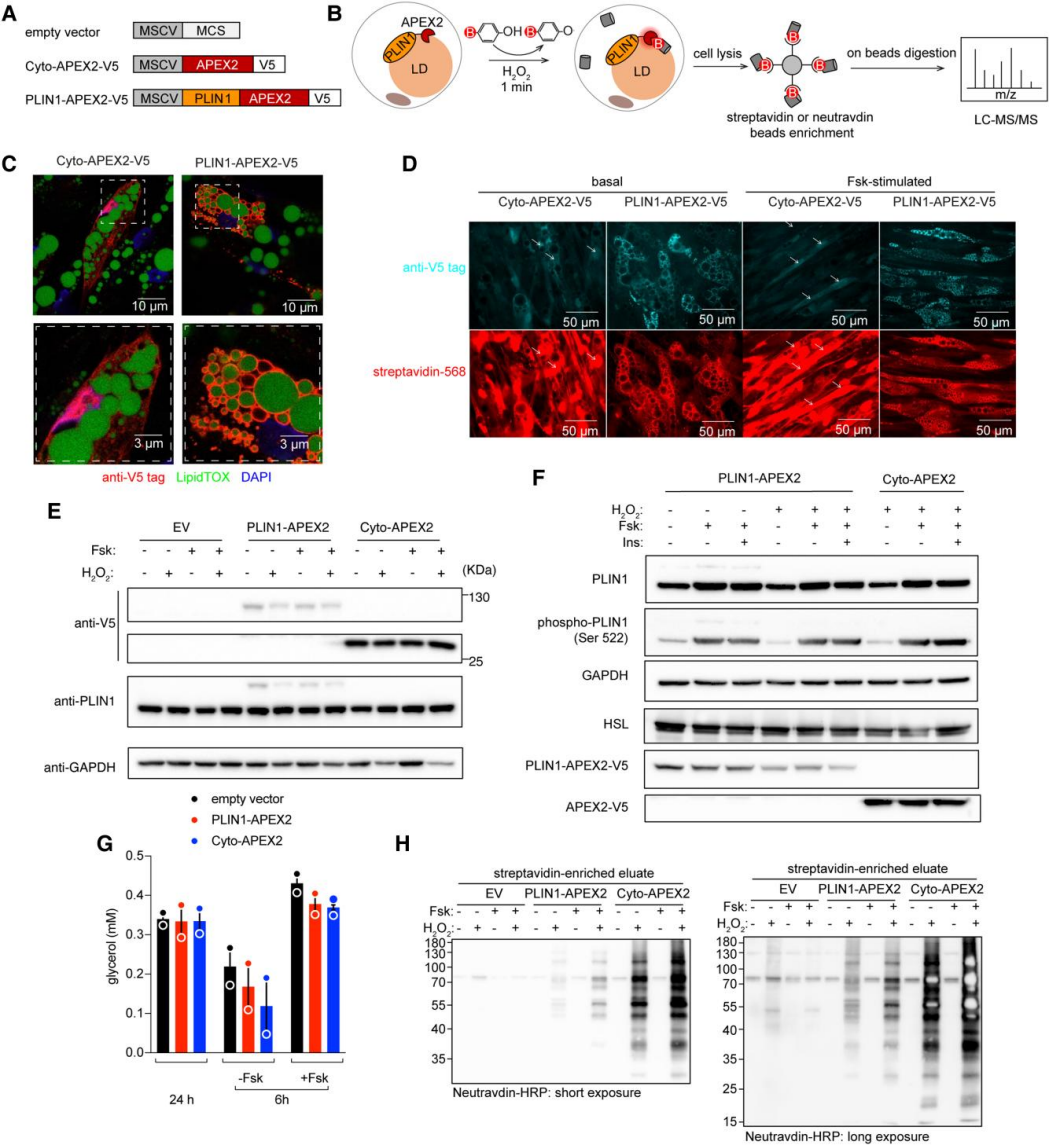
PLIN1-APEX2 fusion construct targets to LDs and maintains enzymatic activity without perturbing lipolysis in human adipocytes. A) Lentiviral vectors used in this study. B) Workflow of PLIN1-APEX2-mediated proximity labeling. C) Images of human primary adipocytes transduced with Cyto-APEX2-V5 and PLIN1-APEX2-V5, stained for V5 epitope tag, LipidTOX, and DAPI as indicated below the panel. Magnified regions highlight the cellular regions with LDs. D) Cells treated with BP/hydrogen peroxide stained for V5 epitope tag (upper panels) and with streptavidin–HRP (lower panels) to detect the biotinylated proteins. E and F) Western blots of whole-cell lysates from cells treated as described above each label, probed with antibodies to the proteins indicated to the left of each band. G) Glycerol concentration in cell medium (error bars = range, *n* = 2, biological replicates). H) Western blot of proteins enriched by streptavidin beads, visualized using neutravidin–HRP for short (left) and long (right) exposures.

Three lentiviral vectors were constructed: a V5-tagged APEX2 genetically fused to the C-terminus of PLIN1 (PLIN1-APEX2-V5), a cytosolic version of APEX2 as a spatial reference control (Cyto-APEX2-V5), and an empty vector served as a negative control (Fig. 1A). Cells transduced with each construct were stimulated with forskolin (Fsk) for 6 h to induce lipolysis, and then incubated with BP for 30 min. H_2_O_2_ was added for 1 min to initiate labeling, after which cells were fixed for immunofluorescence analysis or lysed in a boiling buffer containing 2% SDS (sodium dodecyl sulfate). Biotinylated proteins in the diluted lysates were recovered using streptavidin or neutravidin beads for further identification by LC-MS/MS (liquid chromatography and tandem mass spectrometry) (Fig. 1B). We first determined the subcellular localization of the APEX2 constructs by immunofluorescence using antibodies against V5 tag (Fig. 1C). Cyto-APEX2-V5 was diffusely distributed throughout the cell, including cytoplasm and nucleoplasm. In contrast, PLIN1-APEX2-V5 was localized to the periphery of LDs, which were visualized by LipidTOX staining. To determine whether protein biotinylation would be restricted to the vicinity of the expressed constructs, we visualized the biotinylated proteins using streptavidin Alexa Fluro-568. A diffuse, whole-cell labeling pattern was observed in cells expressing Cyto-APEX2-V5, but biotinylation in cells expressing PLIN1-APEX2-V5 was observed on the periphery of LDs, either in the absence or in the presence of Fsk stimulation (Fig. 1D), confirming that labeling was restricted to the vicinity of each construct.

As the overexpression of proteins can lead to perturbation of their physiological functions, we evaluated the level of expressed constructs. Western blotting of whole-cell lysates using anti-V5 antibody revealed bands at the expected molecular weights of PLIN1-APEX2-V5 (∼90 kDa) and Cyto-APEX2-V5 (∼27 kDa) in cells expressing the corresponding constructs, with significantly more abundant Cyto-APEX2-V5 expression (Fig. 1E). Probing with anti-PLIN1 antibody revealed a band at the expected molecular weight of endogenous PLIN1 (∼60 kDa) in all samples, and an additional, much fainter band corresponding to PLIN1-APEX2-V5 in cells transfected with the corresponding construct. Thus, PLIN1-APEX2-V5 was expressed at much lower levels than endogenous PLIN1. To verify that expressed PLIN1-APEX2-V5 did not functionally interfere with endogenous PLIN1, we measured basal and Fsk-stimulated PLIN1 phosphorylation and lipolysis. We observed similarly enhanced phosphorylation of PLIN1 in response to Fsk in cells expressing both constructs (Fig. 1F). Moreover, PLIN1 phosphorylation was similar in cells expressing PLIN1-APEX2-V5 incubated without or with H_2_O_2_, indicating that the conditions employed during the biotinylation reaction did not interfere with signal transduction (Fig. 1F). To monitor lipolysis, we measured basal glycerol accumulation in the media over 48 h, and glycerol release over 6 h in the presence or absence of Fsk. No difference in glycerol accumulation was observed between cells expressing the empty vector, PLIN1-APEX2-V5 or Cyto-APEX2-V5 (Fig. 1G).

We then probed the western blots with HRP (Horseradish peroxidase)–streptavidin to obtain an overview of all proteins biotinylated by each construct. In lysates of cells transduced with an empty vector, only a few faint bands, possibly corresponding to endogenous biotin-dependent carboxylases were detected. In contrast, a large number of biotinylated proteins were seen in lysates from Cyto-APEX2-V5 and PLIN1-APEX2-V5 transduced cells (Fig. 1H), with significantly more in the former condition.

### Identification of biotinylated proteins by LC-MS/MS

**Fig. 2.**
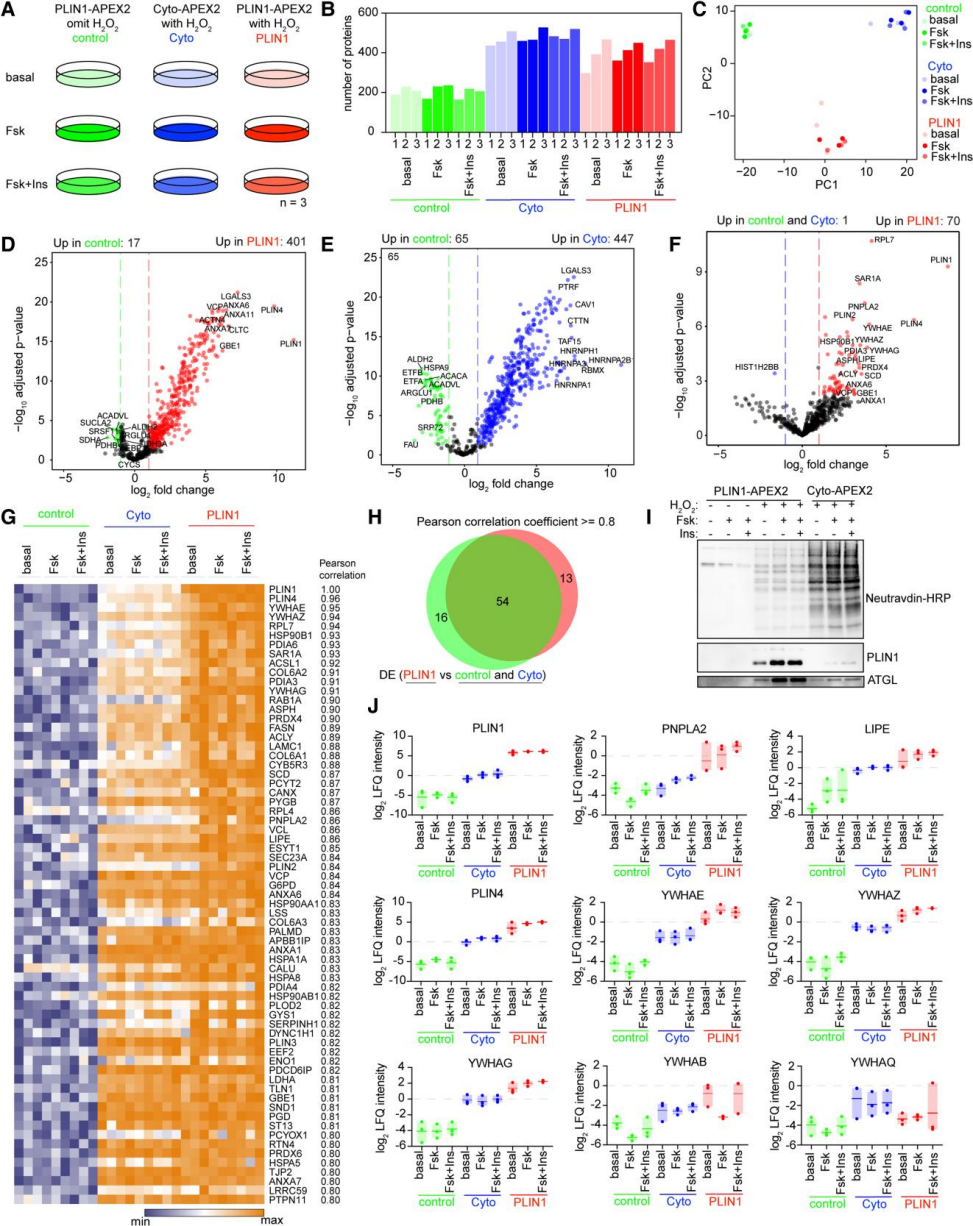
Identification of PLIN1-associated proteins in human adiopocytes. A) Design of labeling and proteomic experiments. The results are derived from three independent experiments. B) Bar graph showing the total number of proteins identified by LC-MS/MS in each sample. C) Scatter plot of the first two principal components of LFQ intensities of proteins identified by LC-MS/MS across all the samples. D and E) Volcano plots of biotinylated proteins enriched across all samples (Basal, Fsk, and Fsk + insulin) by PLIN1-APEX2 (D) or Cyto-APEX2 (E) compared with negative control samples. F) Volcano plot of biotinylated proteins enriched across all samples (Basal, Fsk, and Fsk + insulin) by PLIN1-APEX2 compared with combined negative control and Cyto-APEX2. Colored symbols in D, E, and F correspond to Benjamini–Hochberg-adjusted *P*-value <0.05 and log_2_-fold change ≥1.0. G) Heatmap of proteins enriched by PLIN1-APEX2 based on Pearson correlation coefficient >0.80 with PLIN1 values across all samples. H) Venn diagram showing the overlap between LD proteomes identified by differential enrichment analysis (F) and Pearson correlation analysis (G). I) Western blot analysis using neutravidin–HRP and antibodies against PLIN1and ATGL for proteins enriched by neutravidin beads. J) Box plots of LFQ intensities of indicated proteins in *n* = 3 independent replicate samples.

We obtained biotinylated proteins from cells transduced with PLIN1-APEX2-V5, Cyto-APEX2-V5, or cells transduced with PLIN1-APEX2-V5 but not exposed to H_2_O_2_, therefore preventing biotinylation (negative control). Transduced cells were treated without or with Fsk for 6 h, and one set of cells was treated with insulin for 1 h prior to the addition of Fsk (Fig. 2A). Biotinylated proteins were recovered from three independent sets of transduced cells and identified by LC-MS/MS following on-bead digestion. The number of proteins identified (LFQ [label-free quantification] >0 in >5 out of 9 replicates) was on average 205, 402, and 481 in the negative control, PLIN1-APEX-V5, and Cyto-APEX2-V5 groups, respectively (Fig. 2B and Table S1). Principal component analysis revealed three distinct clusters, but different lipolytic conditions within each cluster (i.e. the presence of Fsk or Fsk + insulin) did not significantly contribute to the variance (Fig. 2C).

Differential enrichment analysis using LFQ intensity values (Benjamini–Hochberg-adjusted *P*-value <0.05 and log_2_-fold change ≥1.0) revealed 401 proteins to be significantly enriched by PLIN1-APEX2-V5 compared with negative control (Fig. 2D and Table S2, Tab 1), with the highest differentially expressed proteins corresponding to PLIN1 and PLIN4. Four hundred and forty-seven proteins were significantly enriched by Cyto-APEX2-V5 relative to negative control (Fig. 2E and Table S2, Tab 2). Seventy proteins were significantly enriched by PLIN1-APEX2-V5 relative to the combined negative control and Cyto-APEX2-V5 samples (Fig. 2F and Table S2, Tab 3). Among these 70 proteins, PLIN1 PLIN2, PLIN4, RAB1A, RAB7A, CYB5R3, PNPLA2, LIPE, VCP, and LSS have been previously reported to be associated with LDs. We searched for proteins specifically enriched by PLIN1-APEX2-V5 in response to Fsk or Fsk + insulin. Unexpectedly, neither Fsk or Fsk + insulin treatment resulted in significant changes in the basal PLIN1 interactome (Figs. S1, S2 and Table S2, Tabs 4–8), suggesting that the regulation of lipid mobilization might be more dependent on the activities of key proteins rather than their abundance on the LD surface. To identify all potential PLIN1-interacting proteins, we analyzed enrichment across all lipolytic conditions. Proteins were ranked by the correlation of their imputed LFQ intensity values with those of PLIN1 (Pearson correlation coefficient ≥0.8; Fig. 2G and Table S3). Proteins correlated with PLIN1 included known LD-binding proteins and multiple additional ER (endoplasmic reticulum) proteins, and largely overlapped with those found by differential enrichment analysis (Fig. 2H). To verify the results obtained by LC-MS/MS, we conducted western blotting of recovered biotinylated proteins and observed significant enrichment of PLIN1 and ATGL by PLIN1-APEX2-V5 (Fig. 2I).

We were intrigued by the enrichment of four members of the 14-3-3 family of proteins (YWHAB, YWHAE, YWHAG, and YWHAZ) by PLIN1-APEX2-V5, as these are adapter/scaffold proteins that bind to phosphorylated targets, regulating their activity, localization, and/or stability. Moreover, YWHAB interaction with PLIN1 was decreased by Fsk (Table S2, Tab 4), and this decrease was reversed by insulin (Table S2, Tab 6), although these interactions did not reach statistical significance upon adjustment for multiple comparisons. We compared the LFQ intensity values of all detected 14-3-3 family proteins (YWHAE, YWHAZ, YWHAG, YWHAB, and YWHAQ) to those of the known LD proteins PLIN1, PNPLA2, LIPE, and PLIN4 (Fig. 2J). Three of these proteins (YWHAE, YWHAZ, and YWHAG) are enriched by PLIN1-APEX2 under all three lipolytic conditions, while YWHAB seemed more variable, being enriched only under basal and Fsk + insulin conditions.

**Fig. 3.**
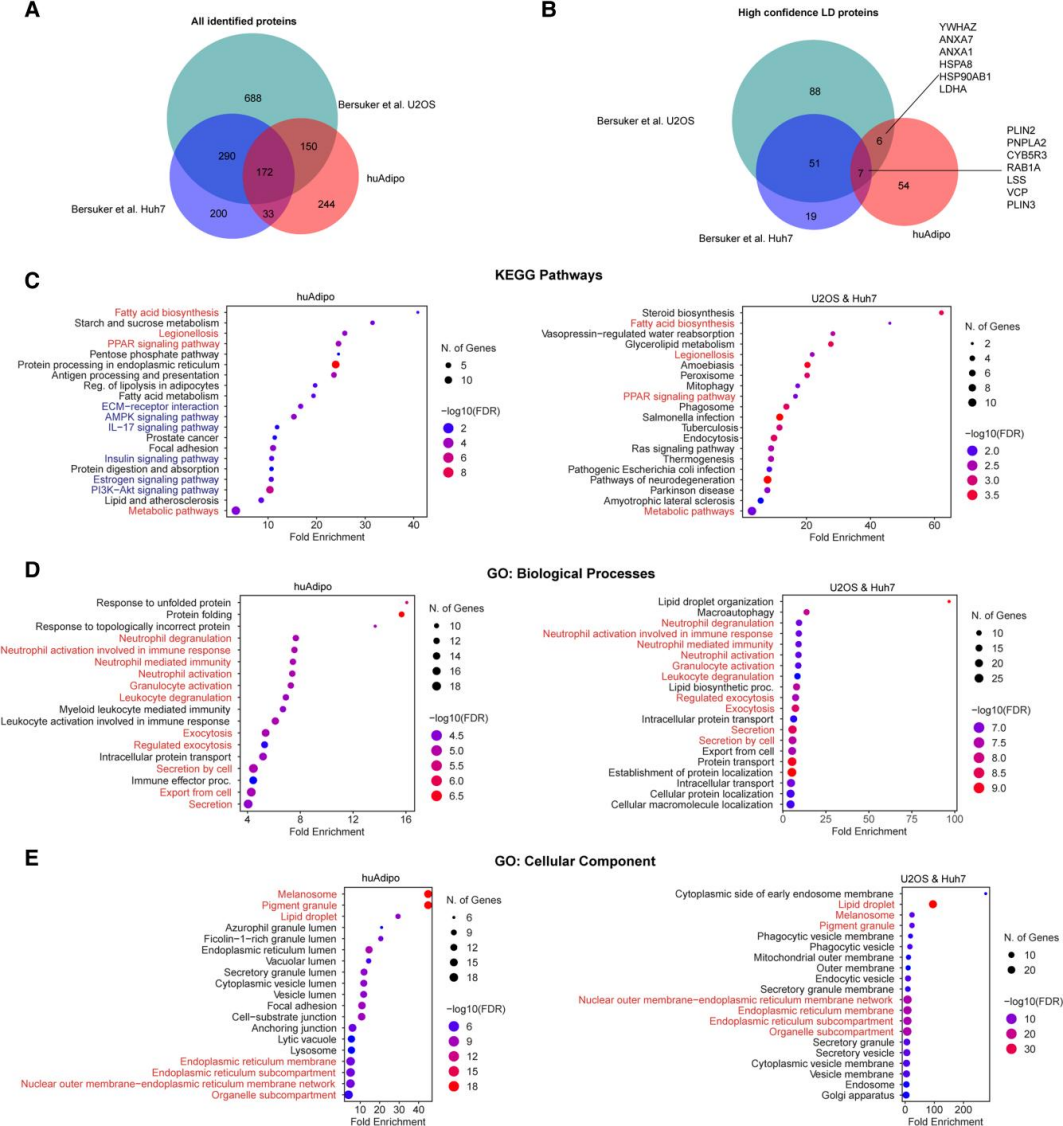
Human primary adipocyte LDs are enriched in elements of hormonal signaling pathways. A and B) Venn diagrams of all proteins (A) and high-confidence LD proteins (B) identified in U2OS and Huh7 by Bersuker et al. (19), and human primary adipocytes. C–E) KEGG pathway analysis (C), GO biological process terms (D), and GO cellular component terms (E), of high-confidence LD proteins from human primary adipocytes (left) and from U2OS/Huh7 cells (right). Pathways in common are highlighted in red, and signaling pathways specific to adipocytes are in blue.

To shed light on the potential biological significance of the PLIN1 interactome in human adipocytes, we contextualized our results by comparing them with those obtained by Bersuker et al. (19), who used APEX2 to identify LD proteins in two cancer cell lines (U2OS and Huh7). Using a combination of PLIN2-APEX2 and ATGL-APEX2, Bersuker et al. identified 1,302 and 699 total biotinylated proteins in U2OS and Huh7 cells, compared with our 599 proteins identified in human adipocytes by PLIN1-APEX2. One hundred and seventy-four proteins were identified in all three cell types (Fig. 3A and Table S4). Of these, Bersuker et al. identified 57 proteins as associated with LDs with high confidence, close in number to the 54 proteins identified in our study (Fig. 2G). Of the high-confidence LD proteins, seven proteins (PLIN2, PLIN3, PNPLA2, RAB1A, CYB5R3, LSS, and VCP) were identified in all three cell types (Fig. 3B and Table S4), suggesting they represent the core machinery necessary for LD assembly.

To distinguish aspects of LD function that might be cell–type specific, we conducted pathway analyses on the high-confidence LD proteins from human adipocytes (Fig. 3, left panels) or U2OS and Huh7 (Fig. 3, right panels). Pathway analysis (KEGG, Kyoto Encyclopedia of Genes and Genomes) showed common enrichment in pathways of fatty acid biosynthesis and PPARγ (Peroxisome Proliferator Activated Receptor Gamma) signaling, but pathways including AMPK (adenylate monophosphate activated protein kinase) signaling, IL-17 (interleukin-17) signaling, and PI3K-Akt (phosphatidylinositol 3-kinase-Akt kinase) signaling were enriched only in adipocyte LDs (Fig. 3C and Table S5, Tabs 1 and 2). Gene ontology (GO) defined biological processes in both cases were enriched for immune cell functions, involving membrane trafficking proteins and chaperones (Fig. 3D and Table S5, Tabs 3 and 4), and consistent with a proposed role for LDs in early innate immune responsiveness (21, 22). Cellular components identified as enriched by high-confidence LD proteins also included the endoplasmic reticulum, lysosomes, and mitochondria (Fig. 3E); 11 proteins detected in human adipocyte LDs are associated with mitochondria, 36 with the endoplasmic reticulum, 28 with the nucleus, and the remainder are cytoplasmic or of indetermined subcellular localization. These results are consistent with the existence of extensive contact sites between LDs and other organelles in all other cell types (23). In aggregate, the contrasting characteristics of LD proteins in human adipocytes vs. cancer cell lines suggest that signaling pathways associated with systemic metabolic control play a more substantial role in the regulation of adipocyte LDs.

### Functional role of 14-3-3 proteins

**Fig. 4.**
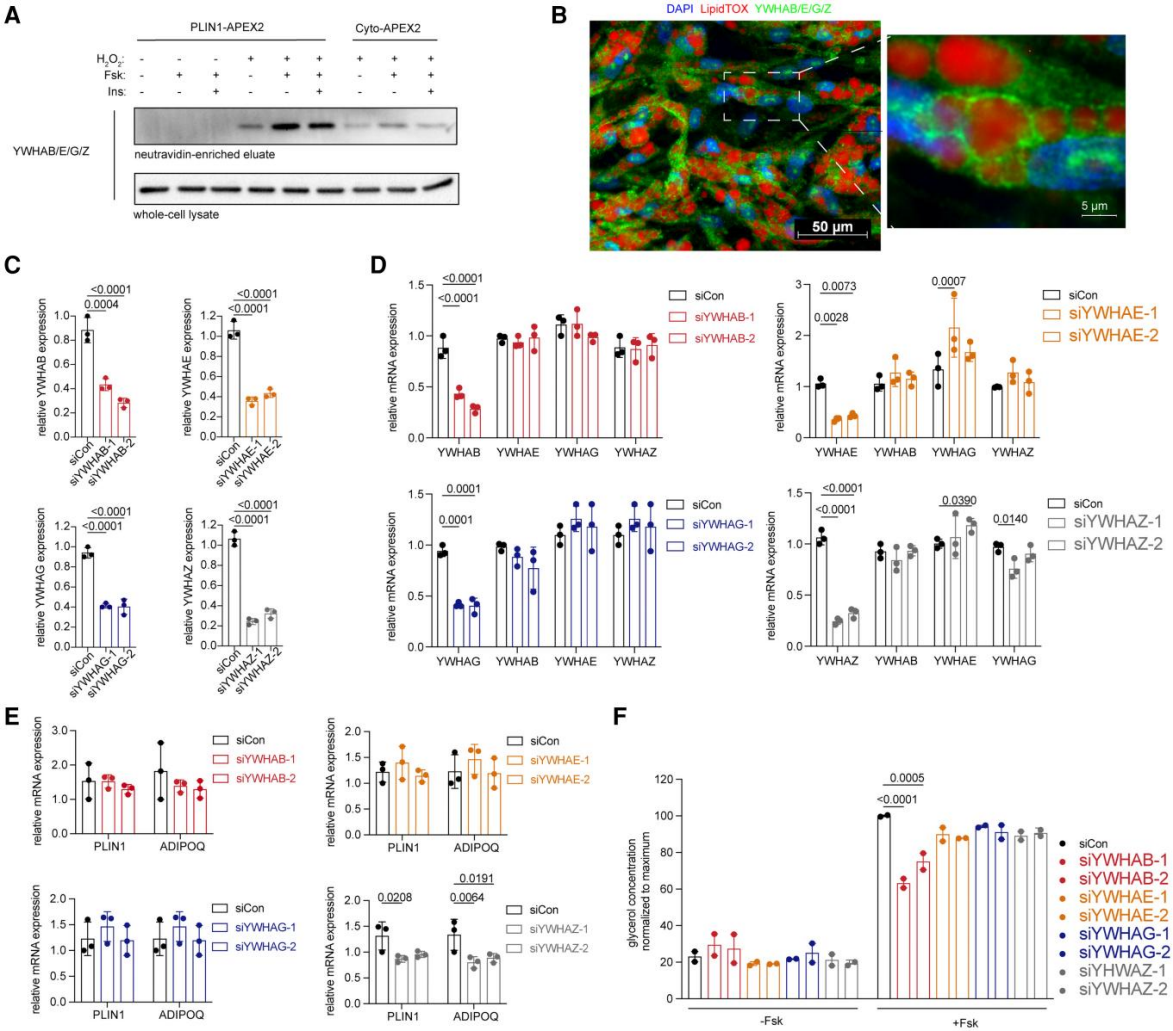
Interactions of 14-3-3 (YWHA) protein with PLIN1 and specific role of YWHAB in Fsk-stimulated lipolysis. A) Western blot probed with antibody recognizing YWHAB/Z/G/E of proteins biotinylated by PLIN1-APEX2 enriched by neutravidin beads compared with whole-cell lysate. B) Immunofluorescence analysis of primary adipocytes using antibody recognizing YWHAB/Z/G/E (green), DAPI (blue), and LipidTOX (red). C) RT-PCR of RNA obtained 48 h after transfection of adipocytes with two siRNAs targeting *YWHAB*, *YWHAE*, *YWHAZ*, and *YWHAG*, as indicated in the *y*-axis. D) RT-PCR of RNA obtained 48 h after transfection of adipocytes transfected with two siRNAs targeting *YWHAB*, *YWHAE*, *YWHAZ*, or *YWHAG*, as indicated in the legend, probed for the transcript indicated in the *x*-axis. E) RT-PCR analysis of RNA obtained 48 h after transfection of adipocytes with siRNAs targeting *YWHAB*, *YWHAE*, *YWHAZ*, and *YWHAG*, probed for expression of *PLIN1* and *ADIPOQ.* For C, D, and E, values are expressed as the fold relative to siRNA control values. Bars represent means and SD of *n* = 3 biological replicates. Statistical differences were assessed using two-way ANOVA, and exact *P*-values are shown. F) Glycerol accumulation over 6 h from cells treated with vehicle or Fsk, 48 h after transfection with siRNAs targeting *YWHAB*, *YWHAE*, *YWHAZ*, and *YWHAG.* Bars represent the mean and SD of *n* = 2 independent experiments, each conducted with 3-fold technical replication. Statistical differences were assessed using two-way ANOVA, and exact *P*-values are shown.

To verify the presence and further explore the function of the 14-3-3 proteins enriched on adipocyte LDs, we first analyzed the specificity of commercially available antibodies for this family of proteins. We generated HA (hemagglutinin)-tagged versions of YWHAB, YWHAG, YWHAZ, and YWHAE, expressed them in 293T cells, isolated the proteins by immunoprecipitation using anti-HA antibodies, and conducted western blotting. Most commercially available antibodies lacked specificity, except for those raised against YWHAG and one against YWHAB (Fig. S3). Western blotting of proteins biotinylated by PLIN1-APEX2-V5 using an antibody that detects YWHAB, YWHAG, YWHAE, and YWHAZ (Abcam) revealed enrichment relative to Cyto-APEX2-V5 under all treatment conditions (Fig. 4A). Immunofluorescence analysis of adipocytes using the same antibody revealed a punctate pattern surrounding the LDs, consistent with close interactions of 14-3-3 proteins with PLIN1 on the LD surface (Fig. 4B).

We then used siRNAs to knock down the expression of each of the LD-enriched 14-3-3 proteins in differentiated adipocytes. We achieved 60–70% knockdown efficiency for each isoform (Fig. 4C), and found that knockdown of the targeted isoforms did not alter the expression level of the nontargeted isoforms (Fig. 4D), consistent with absence of compensatory effects. To determine whether knockdown of these genes would influence adipocyte differentiation, we measured expression levels of PLIN1 and ADIPOQ by RT-PCR (Fig. 3E). While knockdown of YWHAB, YWHAE, and YWHAG had no significant effect, YWHAZ knockdown significantly decreased differentiation (Fig. 4E). To assess specific LD functions, we measured basal and stimulated lipolysis (Fig. 4F). Basal lipolysis was not affected by the knockdown of any of the isoforms, but Fsk-stimulated lipolysis was decreased specifically in response to knockdown of YWHAB (Fig. 4F).

**Fig. 5.**
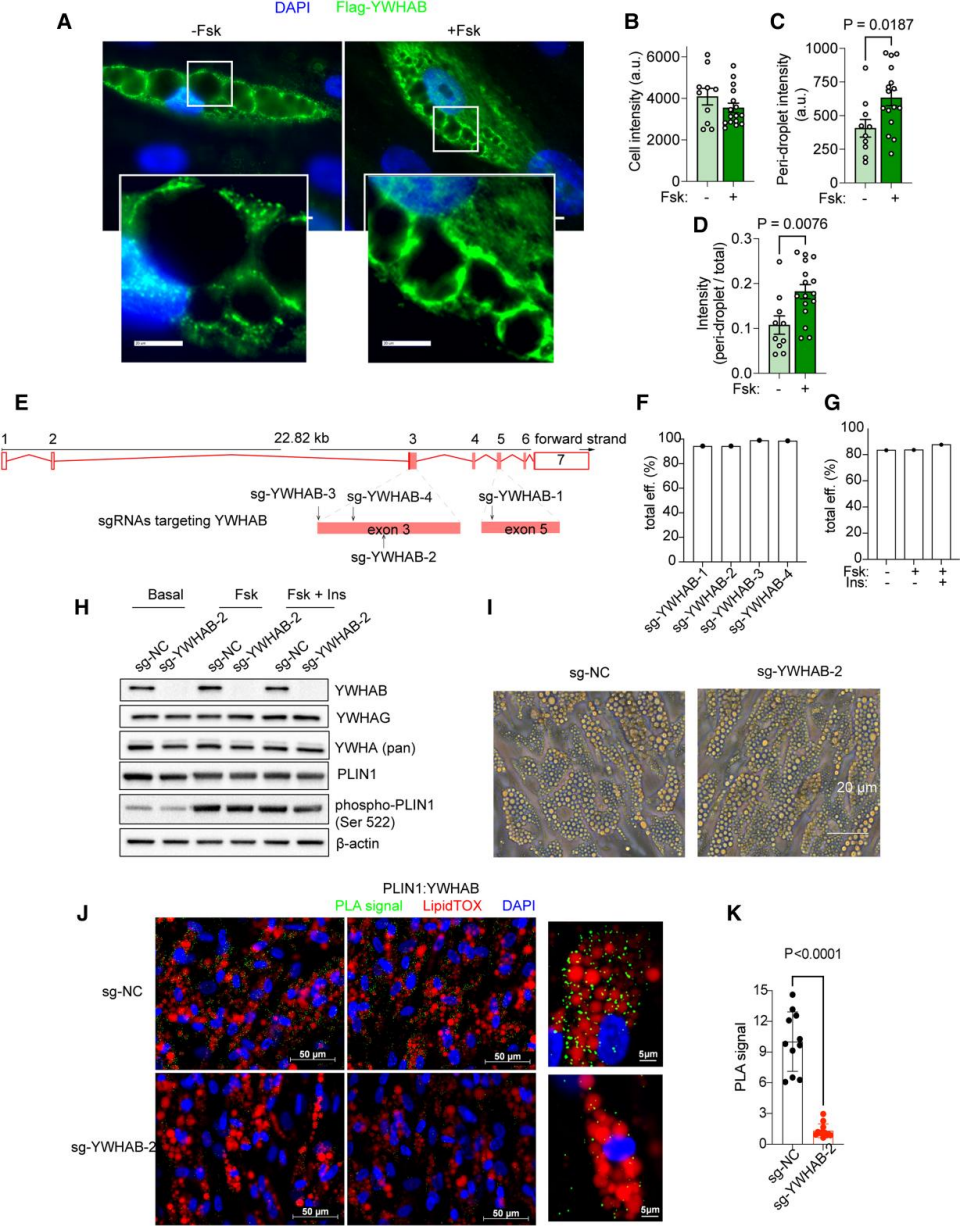
YWHAB is recruited to LDs in response to Fsk but is not required for differentiation or Fsk-stimulated PLIN1 phosphorylation. A) Representative images of adipocytes differentiated from cells electroporated with Flag-tagged YWHAB stained with anti-Flag antibodies (green) and DAPI (blue), in cells treated with vehicle or Fsk. Bars = 20 μm. B–D) Quantification of total (B), peridroplet (C), and ratio of peridroplet/total (D) signal intensity for Flag staining in cells expressing Flag-YWHAB. Plotted are means and SEM for values for *n* = 10 and *n* = 16 cells from cells treated with vehicle or Fsk, respectively. Statistical significance was assessed two-tail Student’s *t*-test, and exact *P*-values are shown. E) Localization of sg-YWHAB-1, sg-YWHAB-2, sg-YWHAB-3, and sg-YWHAB-4 in exons 3 and 5 of the human YWHAB coding region. F and G) Editing efficiency of sgRNAs by different guides (F) and in control or Fsk-treated adipocytes electroporated with sg-YWHAB-2 (G), determined by TIDE analysis. H) Western blots for proteins indicated from adipocytes differentiated from progenitor cells electroporated with nontargeting control (sg-NC) or sg-YWHAB-2 under different lipolytic conditions. I) Representative phase images of human adipocytes without or with YWHAB knockout on day 8 of differentiation. J) Representative images of adipocytes differentiated from cells electroporated with nontargeting control (sg-NC, top panels) or sg-YWHAB-2 (bottom panels), visualizing the proximity ligation product (PLA, green) of antibodies to PLIN1 and YWHAB. Cells are counterstained with DAPI (blue) and LipidTOX (red). K) Mean and SD of total intensities of PLA signal in *n* = 11 independent fields of cells stained as in (J). Statistical difference was assessed using two-tail Student’s *t*-test, and exact *P*-value is shown.

To further explore the subcellular localization of YWHAB and to ensure specificity of immunostaining, we generated and expressed Flag-tagged constructs of the protein. The flag-tagged protein was distributed in a punctate pattern similar to that seen with the pan-YWHA antibody shown in Fig 4B, with signal surrounding LDs and some signal residing within the nucleus (Fig. 5A). After 6 h of Fsk stimulation, the signal intensity around the LDs became more apparent (Fig. 5A, enlarged insets). Quantification of mean pixel intensity showed that the total cellular signal trended to decrease with Fsk (Fig. 5B), but the intensity surrounding the droplets increased (Fig. 5C). This effect was more significant when the ratio of peridroplet intensity was expressed relative to the total cell intensity (Fig. 5D). These results are consistent with a model where YWHAB is recruited to the surface of LDs in response to lipolytic stimulation, into proximity to PLIN1.

To further probe the functional role of YWHAB, we employed CRISPR-Cas9 to achieve full deletion of the protein. To define the optimal genomic locus for YWHAB gene disruption, we designed four sgRNAs: sg-YWHAB-1 targeted exon 5 and sg-YWHAB-2, sg-YWHAB-3, and sg-YWHAB-4 targeted exon 3, both exons within the open reading frame of the YWHAB gene (Fig. 5E). We delivered the RNP (ribonucleoprotein) complexes containing Cas9 and sgRNA to progenitor cells according to a previously developed method (24). Small insertions and deletions were generated by the four different sgRNAs in 85–99% of cells (Fig. 5F), and the deletions were largely maintained upon differentiation of progenitors into adipocytes (Fig. 5G). To determine whether the deletions would result in protein deficiency, we performed western blotting with verified antibodies to YWHAB and, for comparison, YWHAG. While YWHAB was undetectable after CRISPR-Cas9-mediated excision under all lipolytic conditions tested, no difference in YWHAG expression was observed (Fig. 5H). We also noted that neither the abundance of PLIN1 nor cAMP-stimulated signal transduction to PLIN1 was affected by YWHAB deficiency, as the effects of Fsk to stimulate PLIN1 phosphorylation were not impaired (Fig. 5H). The maintenance of PLIN1 levels is consistent with phase imaging of LDs, indicating that YWHAB knockout did not cause impairment of cell differentiation (Fig. 5I). The efficient CRISPR-Cas9 knockdown of YWHAB allowed us to verify its proximity to PLIN1 using proximity ligation (Fig. 5J and K). A clear signal reflecting the proximity of anti-PLIN1 and anti-YWHAB antibodies was detected surrounding the LDs (Fig. 5J, top panels), and this signal was abrogated in cells lacking YWHAB (Fig. 5J, bottom panels), as quantified by image analysis (Fig. 5K).

**Fig. 6.**
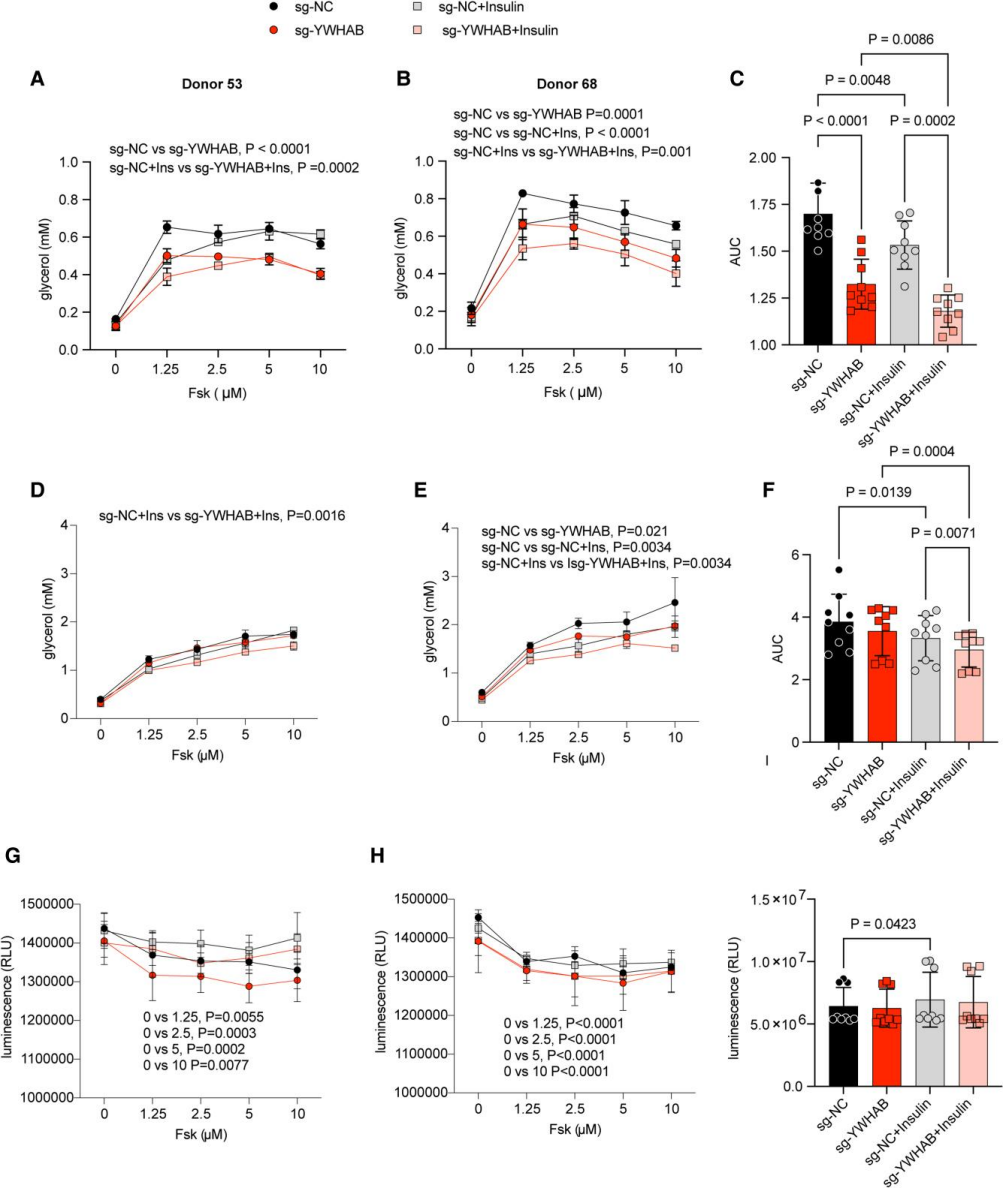
YWHAB deletion mitigates Fsk-stimulated lipolysis through a mechanism distinct from the antilipolytic effect of insulin. Adipocytes differentiated from cells electroporated with nontargeting control (sg-NC) or sg-YWHAB-2 were stimulated with different concentrations of Fsk without or with the addition of insulin. Glycerol accumulation was measured after 6 h (A–C) and 24 h (D–F) of stimulation, and CellTiter-Glo 2.0 assay was performed after 24 h of stimulation (G–I). Plotted are means and SD of values derived from *n* = 3 independent cultures from each donor. Results from two donors are exemplified (donor 53, A, D, G; donor 68, B, E, H). Area under the curve plots are results derived from three independent cultures from each of three donors (donor 53, donor 68, and donor 70, *n* = 9; panels C, F, and I). Statistical significance was determined using two-way (A, B, D, E, G, H) or one-way (C, F, I) ANOVA, and exact *P*-values are shown.

We then examined the functional effects of YWHAB knockout on lipolysis in cells derived from three independent donors (Fig. 6A–F). Fsk induced a dose-dependent increase in glycerol accumulation, resulting from triglyceride lipolysis, which was observed after 6 h (Fig. 6A–C) and persisted for 24 h (Fig. 6D–F) after stimulation. Insulin mitigated the lipolytic effects of Fsk, most prominently at the 6 h time points (Fig. 6A–C). Fsk-stimulated glycerol accumulation was impaired by YWHAB knockout at 6 h (Fig. 6A–C), but was less evident after 24 h (Fig. 6D–F). Notably, the antilipolytic effect of insulin was also seen in cells lacking YWHAB (Fig. 6C and F). To determine whether the effects of YWHAB knockout might be attributable to changes in cell viability, we measured ATP levels (Fig. 6G–I). We find that Fsk treatment decreases ATP levels at all concentrations tested, but insulin mitigates this decrease. However, neither the Fsk-induced decrease in ATP nor its mitigation by insulin was affected by YWHAB knockout (Fig. 6I). These results indicate that YWHAB is required for optimal stimulation of cAMP-induced lipolysis through a pathway independent of insulin signaling.

## Discussion

LDs are found in all cells, where they play a key energetic role by sequestering and releasing lipids for intracellular use. Adipocyte LDs have an additional, key function of meeting the energetic needs of the entire organism. It is therefore of great interest to understand the similarities and differences between nonadipocyte and adipocyte LD function and how specific adipocyte LD mechanisms might operate and impact metabolic health. Prior research has shown that many of the molecular mechanisms controlling LD dynamics are mediated by specialized proteins that target the LD surface, such as the perilipins. In this study, we define proteins that are in close proximity to PLIN1 in human adipocytes, and by comparison to those discovered in other cells by similar approaches, conclude that adipocyte LDs are enriched in proteins associated with hormonal signaling. In particular, multiple isoforms of the 14-3-3 family of proteins are enriched in the vicinity of PLIN1, and we find that one of them, YWHAB is required for cAMP-stimulated lipolysis while maintaining the antilipolytic actions of insulin.

The comparison of proteins interacting with PLIN1 in adipocytes with those interacting with PLIN2 and ATGL in two cancer cell lines provides insight into the core components of LDs. Of all the identified proteins, only seven (PLIN2, PLIN3, PNPLA2, CYB5R3, RAB1A, LSS, and VCP) are found in all three cell types. PLIN1 and PLIN2 are known perilipins, and PNPLA2/ATGL is the major mediator of hormonally stimulated lipolysis. CYB5R3, RAB1A, LSS, and VCP have all been localized to the endoplasmic reticulum, highlighting the close relationship between LD function and biogenesis—indeed, PLIN1 has also been reported to localize to the ER in some studies (25), and LDs and ER maintain close contacts (23). Beyond these seven core common LD proteins, many others are associated with lipid synthetic pathways, as expected given the core function of LDs as reservoirs of triglycerides and other lipids. Interestingly, LD proteins also include chaperones and trafficking proteins that operate in exocytosis, which are enriched in immune cell pathways. These associations not only suggest that LDs may have an important role in immune cell function but also suggest that exocytosis of lipids via vesicular trafficking may be a central, less recognized role of LDs (26). In addition to proteins common to multiple cell types, our study identifies proteins more specifically associated with adipocyte LDs, mapping to the PI-3 kinase, AMPK, insulin, estrogen, and IL-17 signal transduction pathways. These results indicate that adipocyte LDs are poised to respond to hormonal signaling to rapidly meet systemic energy needs.

While proximity labeling is more specific than traditional biochemical methods and capable of capturing LD proteome in intact cells, there are still some limitations in our study. Not all previously reported LD proteins, notably ABHD5 (27), were identified by PLIN1-APEX2 labeling, nor by PLIN2-APEX2 by Bersuker et al. (19). Moreover, we could not determine statistically significant differences in proteins labeled by PLIN1 under different lipolytic conditions, despite clear effects on glycerol release. The absence of major differences may be attributable to our use of label-free proteomics, which may be less quantitative compared with other techniques such as stable isotope labeling by amino acids in cell culture (18) or tandem mass tags (28). Alternatively, lipolysis may be more dependent on the activities of LD proteins, and less on architectural rearrangements of the LD proteome. Despite these limitations, our analysis revealed significant enrichment by PLIN1-APEX2 of proteins implicated in signal transduction, such as YWHA (14-3-3) proteins.

The mammalian YWHA/14-3-3 protein family is comprised of seven isoforms encoded by distinct genes. They preferentially exist as homodimers but can also heterodimerize; each monomer binds to serine/threonine phosphopeptide motifs with an ordered affinity ranking (29), protecting from dephosphorylation. By binding two phosphopeptides, 14-3-3 dimers can change the conformation of a phosphoprotein or mediate its interaction with other phosphoproteins, thereby modulating localization, enzymatic activity, and multiple cellular functions (30). A major role of 14-3-3 proteins in the maintenance of cellular architecture has recently been proposed (31).

In the context of adipocyte function, 14-3-3 proteins have been reported to interact with ACACA, ACLY, and FASN (32), suggesting a key role in the regulation of fatty acid biosynthesis. Interestingly, YWHAB has been implicated in insulin signaling, through interactions with the insulin receptor, IRS1, and phosphodiesterase 3B (12, 33, 34). In our study, complete ablation of YWHAB in human adipocytes did not impair, and indeed was additive, to insulin-mediated suppression of lipolysis, indicating that YWHAB is not required in the insulin signaling pathway to suppress lipolysis.

The enrichment of four isoforms (YWHAE, YWHAZ, YWHAG and YWHAB) in the vicinity of PLIN1, as we infer from their biotinylation by PLIN1-APEX2-V5, is consistent with a major role of phosphorylation/dephosphorylation reactions in the regulation of LD function. Current models of lipolysis activation postulate that cAMP-dependent PKA is indispensable for activation of PLPLA2/ATGL by ABHD5, which is sequestered by PLIN1 under basal conditions and released following PLIN1 phosphorylation. Additionally, ATGL and HSL are also phosphorylated. The timing of these events, and their precise localization on the LD surface, may be coordinated by the actions of the YWHA proteins in the vicinity of PLIN1. A role for YWHA proteins in the actions of perilipins is also consistent with the prior identification of YWHAQ and YWHAZ by proximity labeling with PLIN2 (19) and of YWHAZ/G/E/Q though biochemical methods (35, 36). Our results advance our understanding of the functional role of YWHAB by demonstrating its requirement for maximal cAMP-stimulated lipolysis and its independence from the antilipolytic effects of insulin. Further studies will be required to identify the specific molecular interactions mediated by YWHAB and how these interactions may advance the development of therapeutic interventions for metabolic diseases.

## Materials and methods

### Primary progenitor cells

Progenitor cells were obtained and expanded according to previously published methods (18), from patients undergoing panniculectomy procedures at University of Massachusetts Medical Center with the approval of University of Massachusetts Institutional Review Board (#14734_13). Informed consent was obtained from all participants. Tissue explants were embedded in MatriGel (Corning, cat# 356231; 200 1 mm^3^ explants per 10 cm dish) and cultured for 14 days. Progenitor cells sprouting from the explants were obtained using Dispase followed by Trypsin-EDTA and Collagenase I treatment and expanded in 15-cm cell culture dishes. To induce adipogenic differentiation, progenitor cells at 100% confluency were maintained in DMEM (Dulbecco's modified Eagle's medium) + 10% FBS (fetal bovine serum) supplemented with 0.5 mM 3-isobutyl-1-methylxanthine, 1 µM dexamethasone, and 1 µg/mL insulin for 72 h. Subsequently, half of the medium was replaced with DMEM + 10% FBS every other day for 3 days. On day 8 of differentiation, cells were treated with forskolin (10 µM) without or with insulin (5 µg/mL) for 6 h.

### PLIN1-APEX2-V5 and Cyto-APEX2-V5 constructs

PLIN1 and APEX2-V5 fragments were amplified by PCR from PLIN1 cDNA ORF clone in pcDNA3.1^+^/C-(K)-DYK vector (GenScript: OHu22113) and Twinkle-APEX2-V5 vector (Addgene, plasmid #129705), respectively. PLIN1-APEX2-V5 construct was generated by assembling PLIN1 and APEX2-V5 fragments into the lentiviral vector pCDH-MSCV-MCS-EF1-GFP by HiFi DNA assembly with GGGGS as a fusion protein linker. Cyto-APEX2-V5, HA-tagged YWHA proteins, and Flag-tagged YWHAB were similarly assembled. Primers used for PCR amplification and sequencing of constructs used for proximity labeling are listed in Table 1.

**Table 1.** Primers used for PCR amplification and sequencing of constructs used for proximity labeling.

Primer name	Sequence (5′-3′)
*HiFi DNA assembly primers for PLIN1-APEX-V5 construct*	
PLIN1-FWD	CCCTGGATACCCGTATgccaccatggcagtcaacaaag
PLIN1-REV	AGAGCCACCTCCGCCgctcttcttgcgcagctgg
PLIN1-APEX2-V5-FWD	GGCGGAGGTGGCTCTggaaagtcttacccaactgtgag
PLIN1-APEX2-V5-REV	ttaggtgctgtccaggcc
MSCV-PLIN1-FWD	cctggacagcacctaaGAAGGATCTGCGATCGCTC
MSCV-PLIN1-REV	ATACGGGTATCCAGGGGACAC
*HiFi DNA assembly primers for Cyto-APEX-V5 construct*	
Cyto-APEX2-V5-FWD	ACCCGTATgccaccatgggaaagtcttacccaactgtgag
Cyto-APEX2-V5-REV	ttaggtgctgtccaggcc
MSCV-Cyto-FWD	cctggacagcacctaaGAAGGATCTGCGATCGCTC
MSCV-Cyto-REV	catggtggcATACGGGTATCCAGGGGACAC
*Sequencing primer*	
MSCV-FWD	TCTGTTTGTGTGCTTCTGCTCCCTG

### Lentivirus production, transduction, and labeling

Lenti-X 293T cells were transfected at ∼70% confluency with the lentiviral vector pCDH-MSCV-MCS-EF1-GFP containing gene of interest (18 µg), packaging plasmid psPAX2 (13.5 µg), and envelope plasmid pMD2.G (4.5 µg), using calcium phosphate transfection. Forty-eight and 72 h after transfection, the medium was centrifuged at 500 × *g* for 10 min, filtered through a 0.45-mm filter and concentrated using Lenti-X concentrator and titrated by abm qPCR lentivirus titer kit.

On day 5 of differentiation, cells were transduced at a MOI (multiplicity of infection) of 50 in the presence of 8 µg/mL polybrene. Twenty-four hours after transduction, the lentiviral supernatant was replaced with fresh complete DMEM, and APEX labeling was performed on day 8 of differentiation. To initiate APEX2 labeling, cells were incubated with 3 mL of 500 µM BP in complete DMEM (containing the corresponding amounts of vehicle, Fsk or Fsk + insulin) for 30 min at 37°C. H_2_O_2_ was then added to a final concentration of 1 mM for exactly 1 min at room temperature with gentle agitation. The reaction was stopped by washing cells three times with PBS containing 5 mM Trolox, 10 mM sodium ascorbate, and 10 mM sodium azide. The negative controls which are cells expressing PLIN1-APEX2-V5 were treated identically, except for the 1 min H_2_O_2_ addition step.

### Immunofluorescence staining

Cells were fixed with 4% paraformaldehyde in PBS for 15 min at room temperature, washed with PBS, permeabilized and blocked in PBS containing 1% BSA and 0.3% Triton X-100 for 30 min at room temperature and incubated with primary antibody in permeabilization and blocking buffer overnight at 4°C. Secondary antibodies conjugated to Alexa Fluor-568 or Alexa Fluor-647 were used at 1:1,000 dilution for 1 h at room temperature. To stain LDs, LipidTOX Deep Red Neutral Lipid Stain or LipidTOX Green Neutral Lipid Stain (1:200 dilution) were used in PBS for 30 min. For nuclear staining, Hoechst 33342 was used in permeabilization and blocking buffer for 5 min. Coverslips were mounted using Prolong Gold Antifade Mountant and imaged using Zeiss LSM 800 or Zeiss Axiovert 200M Florescence microscope. Images were analyzed using ImageJ (FIJI).

In situ proximity ligation assays (PLAs) were performed using Duolink in situ red starter kit (MilliporeSigma, DUO92101) in cells incubated with anti-PLIN1 (1:200 dilution, Cell Signaling Technology, #9349) and anti-YWHAB (1:100 dilution, Santa Cruz Biotechnology, sc-25276). Images were taken under ZEISS Axiovert 200M Fluorescence microscope. Quantification was performed using ImageJ (FIJI) software.

### Immunoblotting

After APEX2 labeling, cells were lysed in boiling 2% SDS solution with protease and phosphatase inhibitors. Cell lysates were sonicated and incubated at 37°C for 20 min, followed by another incubation at 65°C for 5 min. Protein concentrations were determined and 40 µg of whole-cell lysates were separated on SDS–PAGE (polyacrylmide gel electrophoresis) gels. Proteins were transferred to PVDF (polyvinylidene difluoride) membranes and blocked with PBST (phosphate-buffered saline with Tween 20) containing 5% skim milk. Primary antibodies were incubated at 4°C overnight and detected with HRP-conjugated secondary antibodies. For identification of biotinylated proteins, membranes were incubated with neutravidin–HRP. HRP activity was visualized using SuperSignal West Pico PLUS substrate and Bio-Rad ChemiDoc imaging system. The following antibodies and reagents were used: anti-V5 tag, anti-PLIN1, anti-phospho-PLIN1-Serine 522, anti-GAPDH, anti-HSL, anti-ATGL, Pierce high-sensitivity neutravidin–HRP, goat antirabbit HRP-conjugated secondary antibody, and goat antimouse HRP-conjugated secondary antibody.

### Enrichment of biotinylated proteins

To enrich biotinylated proteins, 1,800 µg of protein samples in 2% SDS solution were diluted 1:10 with RIPA (radioimmunoprecipitation assay buffer) lysis buffer. Two hundred microliters of Cytiva SpeedBeads magnetic neutravidin-coated particles were washed 2× with RIPA lysis buffer and added to the diluted protein samples, followed by overnight incubation at 4°C with rotating. Beads were washed 4× with RIPA buffer and resuspended in 170 µL of RIPA buffer. One hundred and fifty microliters of bead suspensions were sent for LC-MS/MS analysis and 20 µL were used for elution of biotinylated proteins in 60 µL of 3× Laemmli buffer containing 2 mM biotin and 20 mM DTT (dithiothreitol) with heating at 95°C for 10 min. Thirty microliters of eluates were loaded on SDS–PAGE gels for western blot analysis using neutravidin–HRP or antibodies indicated in each figure. The RIPA lysis buffer was composed of 50 mM Tris-HCl, pH 7.5, 150 mM NaCl, 0.5% sodium deoxycholate, 1% Triton X-100 in distilled water. The washing buffer was composed of 50 mM Tris-HCl, pH 7.5, 150 mM NaCl, 0.1% SDS, 0.5% sodium deoxycholate, and 1% Triton X-100 in distilled water.

### On-bead digestion and LC-MS/MS analysis of biotinylated proteins

Bead suspensions were sent to Proteomics and Mass Spectrometry Facility at University of Georgia for proteomic analysis. The beads were washed with 200 µL of 20 mM ammonium bicarbonate, vortexed, and centrifuged at 1,000 × *g* for 2 min. The solvent was removed and replaced with 20 mM ammonium bicarbonate. This wash step was repeated five more times. The proteins on the beads were then digested with 0.2 µg of sequencing-grade trypsin in about 40 µL of bicarbonate buffer overnight at room temperature. Next day, 100 µL of water were added to quench trypsin digestion and then the tryptic peptides in the supernatant were collected. The peptides were dried down in a vacufuge and resuspended in 10 µL of 2% acetonitrile containing 0.1% formic acid for LC-MS/MS. Samples were analyzed on a Thermo Fisher LTQ Orbitrap Elite Mass Spectrometer coupled with a Proxeon EASY-nLC system. Briefly, 1 µL of enzymatic peptides was loaded into a reversed-phase column (self-packed 100 µm ID column with 200 Å 5 µM Bruker MagicAQ C18 resin, ∼15 cm long), then directly eluted into the mass spectrometer at a flow rate of 450 nL/min. The two-buffer gradient elution (0.1% formic acid as buffer A and 99.9% acetonitrile with 0.1% formic acid as buffer B) starts with 2% B, holds at 0% B for 2 min, then increases to 30% B in 60 min, to 50% B in 10 min and to 95% B in 10 min. The data-dependent acquisition method was used to acquire MS data. A survey MS scan was acquired first, and then the top 10 ions in the MS scan were selected for following CID MS/MS analysis. Both MS and MS/MS scans were acquired by Orbitrap at resolutions of 120,000 and 15,000, respectively.

### Mass spectrometry

MaxQuant (version 1.6.5.0) algorithm (37) was used for label-free quantification of proteins and peptides. The raw data were searched against the UniProt human proteome database (UP000005640) with carbamidomethyl cysteine as a fixed modification and variable modifications, including N-terminal protein acetylation and oxidized methionine. Trypsin specificity allowed cleavages N-terminal to proline and a maximum of two missed cleavages. First search peptide tolerance was 20 ppm, and for the main search, 6 ppm. FTMS (Fourier Transform Mass Spectrometry) MS/MS match tolerance was 20 ppm, and the top MS/MS peaks per 100 Da were set to 12. The minimum peptide length was 7. PSM (peptide-spectrum match) and protein false discovery rates were set to 1%. Protein quantification was based on unmodified, N-terminally acetylated peptides, and peptides with oxidized methionine.

ProVision (38) (https://provision.shinyapps.io/provision/) was used for statistical analysis using LFQ intensity values converted to log_2_ scale and subtracted by the median. Samples were grouped by conditions and the proteins with at least two values per replicate in at least one group were retained for later statistical analysis. Missing values were imputed using the “Missing not At Random” method. The limma package from R Bioconductor was used to identify differentially expressed proteins for each pair-wise comparison using an adjusted *P*-value of 0.05 (Benjamini–Hochberg method) and a |log_2_-fold change| of 1. Heatmaps were generated using the interactive tool Morpheus (Broad Institute). Area-proportional Venn diagram was generated using BioVenn (39). KEGG pathway and GO analysis were performed and visualized using ShinyGO 0.77 (http://bioinformatics.sdstate.edu/go/).

### siRNA-mediated knockdown of 14-3-3 proteins

Three Silencer Select siRNA oligos (Thermo Fisher, siRNA ID: s14961, s14962, s14963) were used for each gene. Transfections were performed on day 6 of differentiation using Endo-Porter (Gene Tools). Analysis was performed 48 h after transfection. The sequences of siRNAs are listed in Table 2.

**Table 2.** Sequences of siRNAs used in this study.

siRNA name	siRNA ID	Sense sequence (5′-3′)	Antisense sequence (5′-3′)
siYWHAZ-1	s14972	GUCUGUAACUGAGCAAGGAtt	UCCUUGCUCAGUUACAGACtt
siYWHAZ-2	s14971	CUCAGUUGCUUAUAAAAAUtt	AUUUUUAUAAGCAACUGAGag
siYWHAE-1	s200463	GGCAAAUGGUUGAGACUGAtt	UCAGUCUCAACCAUUUGCCga
siYWHAE-2	s17	GAAGACAAGCUAAAAAUGAtt	UCAUUUUUAGCUUGUCUUCtc
siYWHAG-1	s14966	AUAACUACCUGAUCAAGAAtt	UUCUUGAUCAGGUAGUUAUcc
siYHWAG-2	s14964	GGGUCAUCAGUAGCAUUGAtt	UCAAUGCUACUGAUGACCCtc
siYWHAB-2	s14962	GGCUUACCAGGAGACAUUtt	AAAUGCUUCCUGGUAAGCCtg
siYWHAB-3	s14963	CGCUGAAUGAAGAGUCUUAtt	UAAGACUCUUCAUUCAGCGta

### RT-qPCR analysis

Total RNA was purified using Direct-zol RNA Miniprep Kit (Zymo Research). Reverse transcriptions were performed for 1 µg RNA using iScript cDNA Synthesis Kit (Bio-Rad). qPCRs were performed with iQ SYBR Green Supermix (Bio-Rad) using CFX96 Touch Deep Well Real-Time PCR Detection System (Bio-Rad). qPCR primers used in this study are listed in Table 3.

**Table 3.** qPCR primers used in this study.

Gene name	Primer orientation	Primer sequence (5′-3′)
YWHAB	FWD	GGCAAAGAGTACCGTGAGAAG
YWHAB	REV	CTGGTTGTGTAGCATTGGGAATA
YWHAE	FWD	GATTCGGGAATATCGGCAAATGG
YWHAE	REV	GCTGGAATGAGGTGTTTGTCC
YWHAZ	FWD	CCTGCATGAAGTCTGTAACTGAG
YWHAZ	REV	GACCTACGGGCTCCTACAACA
YWHAG	FWD	AGCCACTGTCGAATGAGGAAC
YWHAG	REV	CTGCTCAATGCTACTGATGACC
RPL4	FWD	GCCTGCTGTATTCAAGGCTC
RPL4	REV	GGTTGGTGCAAACATTCGGC
PLIN1	FWD	ACCAGCAAGCCCAGAAGTC
PLIN1	REV	CATGGTCTGCACGGTGTATC
ADIPOQ	FWD	TGCTGGGAGCTGTTCTACTG
ADIPOQ	REV	TACTCCGGTTTCACCGATGTC

### CRISPR-Cas9 deletions

RNP complexes of 4 µM sgRNA (IDT or Synthego) and 3 µM SpyCas9 protein (PNA Bio) were prepared in Buffer R provided by the Neon Transfection System Kit to a volume of 6 µL. Progenitor cells were trypsinzed, washed, and resuspended in Buffer R to a density of 2 × 10^5^ cells per 6 µL. Equal volumes of RNP mix and cell suspension were mixed using 10 µL Neon tip and electroporated using the optimized parameters (voltage: 1,350 V, width of pulse: 30 ms, number of pulse: 1). After electroporation, the cells were plated immediately in 12-well plates containing 1 mL of complete media, grown to confluence, and differentiated. sgRNAs used in this study are listed in Table 4.

**Table 4.** sgRNAs targeting YWHAB.

sgRNA name	sgRNA ID	Sequence (5′-3′)
sg-YWHAB-1	Hs.Cas9.YWHAB.1.AA	CACTGTGTCGAACTCCCAGC
sg-YWHAB-2	Hs.Cas9.YWHAB.1.AC	TGACACGCCAGGAAGAGCGG
sg-YWHAB-3	YWHAB + 44901538	CTTGTTCTAGGGAATGACAA
sg-YWHAB-4	YWHAB + 44901616	TGATATGGCTGCAGCCATGA

Genomic DNA was isolated using QuickExtract DNA Extraction Solution (Lucigen). PCRs were conducted with 50 ng of genomic DNA and following primer pairs spanning the sgRNA target site in KAPA HiFi HotStart ReadyMix (Roche). The PCR products were purified by QIAquick PCR Purification Kit and summitted Genewiz for Sanger Sequencing. The control and sample sequence data files were analyzed using TIDE webtool (htEtps://tide.nki.nl/#about). PCR primers designed for sgRNAs targeting YWHAB are listed in Table 5.

**Table 5.** PCR primers designed for amplification of sgRNAs targeted regions of YWHAB.

sgRNA	Primer orientation	Primer sequence
sg-YWHAB-1	FWD	CCACAGACCTAGGGAGTAAGA
sg-YWHAB-1	REV	CAATGTATAACCAAGGCCCAAAG
sg-YWHAB-2	FWD	CCAGATGGGTTGGAAGAAGATT
sg-YWHAB-2	REV	TCAGACCAAAGCAGGACTTTAC
sg-YWHAB-3	FWD	CCCTCCATTTCTTCACCCATAA
sg-YWHAB-3	REV	CTGGCGATGACATCACTACTC
sg-YWHAB-4	FWD	CTGGGACTGTTGGGAAGATTAC
sg-YWHAB-4	REV	TCAGACCAAAGCAGGACTTTAC

### YWHA antibody validation

Lenti-X 293T cells were transfected with pCDNA3.1 plasmids expressing HA-tagged YWHA isoforms using Lipofectamine 3000 (Thermo Fisher Scientific). Cell lysates in 2% SDS were diluted 1:10 with RIPA buffer and sonicated. One microgram protein lysate was immunoprecipitated with monoclonal anti-HA agarose (Millipore) and analyzed by western blotting using the following antibodies: anti-HA Tag (Cell Signaling Technology, #3724), anti-YWHA (pan, Cell Signaling Technology, #8312), YWHAB (Abcam, ab15260), YHWAB (Santa Cruz Biotechnology, sc-25276), YWHAE (Cell Signaling Technology, #9635), YWHAE (Santa Cruz Biotechnology, sc-23957), YWHAZ (Santa Cruz Biotechnology, sc-293415), YWHAZ (Abcam, ab51129), YWHAG (Cell Signaling Technology, #5522S), YWHAG (Novus Biologicals, NB100-407SS), and YWHAG (Santa Cruz Biotechnology, sc-398423).

### Other assays

CellTiter-Glo 2.0 cell viability assay (Promega, Cat# G9243) was employed.

## Supplementary Material

pgad420_Supplementary_Data

## Data Availability

The mass spectrometry proteomic data have been deposited with the ProteomeXchange Consortium via the PRIDE (40) partner repository with the dataset identifier PXD047378. LFQ data are provided in this manuscript. All reagents generated for this project will be made available on request.

## Preprints

This this manuscript was posted on a preprint: https://doi.org/10.1101/2023.05.01.538914.
