# Supplementary material for: Regulation of lipolysis by 14-3-3 proteins on human adipocyte lipid droplets
Source: PNAS Nexus. 2023 Dec 6;2(12):pgad420. doi: 10.1093/pnasnexus/pgad420 (PMC10733194; doi:10.1093/pnasnexus/pgad420)
Supplement: pgad420_Supplementary_Data [file pgad420_supplementary_data.zip › PNASNEXUS-PNASNEXUS-2023-00479R-s06.docx]

**
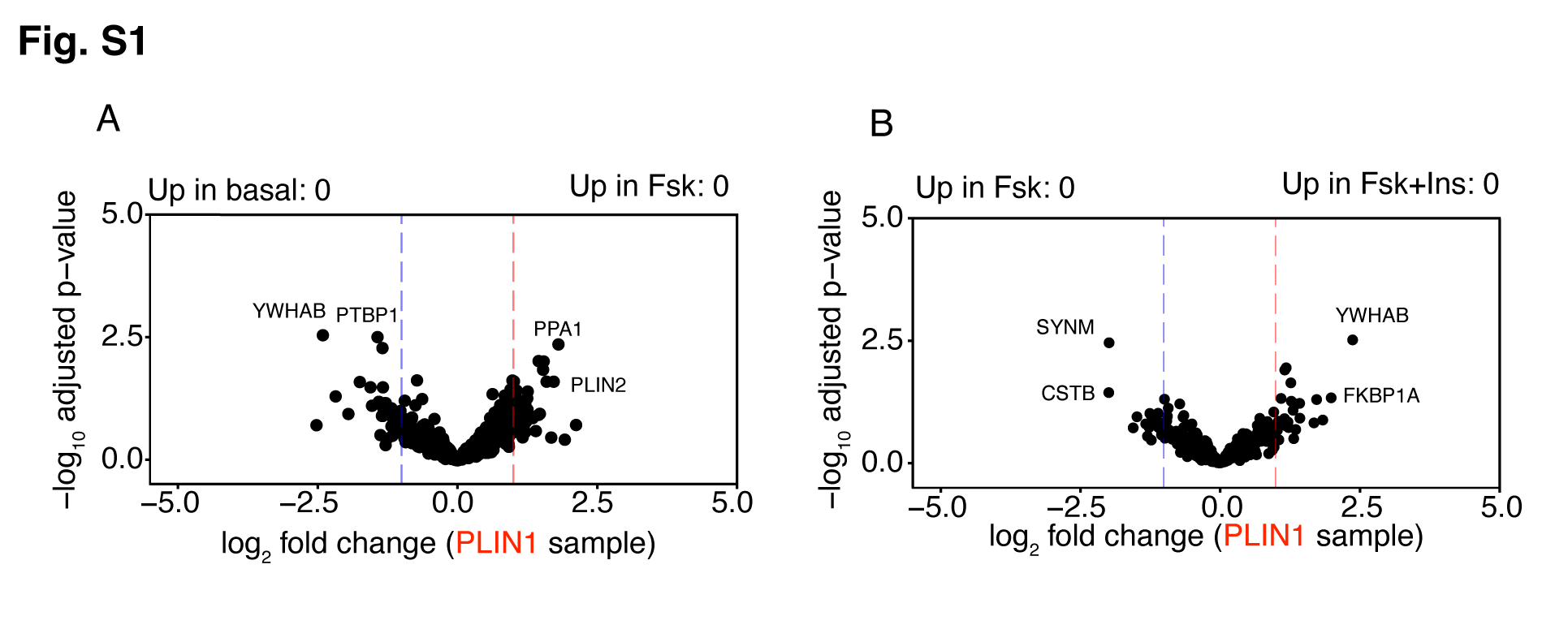
Figure S1. No statistically significant difference in abundance of proteins biotinylated by PLIN1-APEX2 under different lipolytic conditions.** Volcano plots of biotinylated proteins enriched by PLIN1-APEX2 under basal or Fsk-stimulated conditions (A), or under Fsk or Fsk+Ins conditions (B). Statistical significant changes were not detected using Benjamini-Hochberg-adjusted p-value < 0.05 and log2 fold change ≥ 1.0.

**
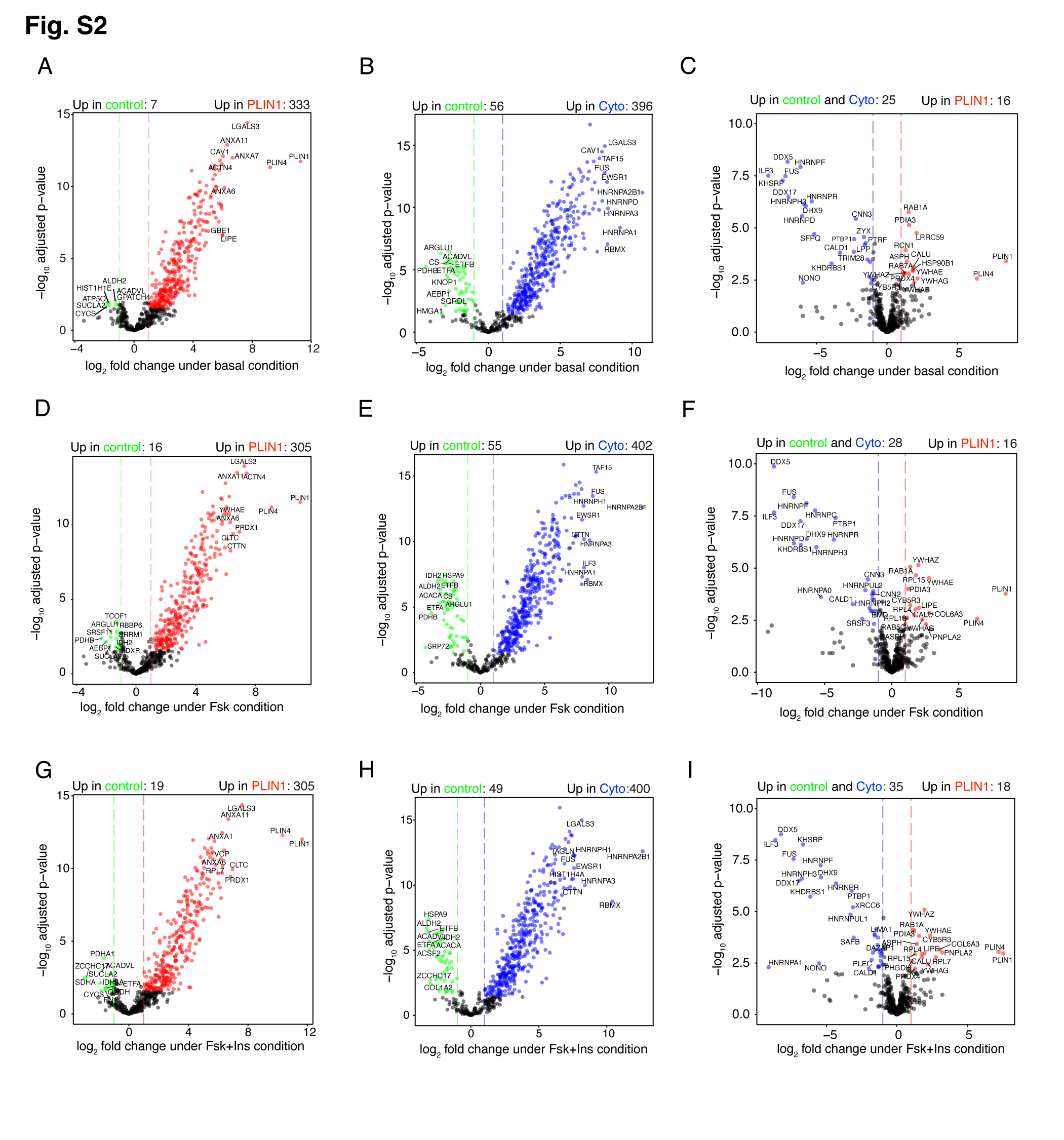
**

**Figure S2. Similar enrichment of biotinylated proteins under different lipolytic conditions.** Volcano plots of biotinylated proteins enriched by PLIN1-APEX2 (A,D,G) or Cyto-APEX2 (B,E,H) compared to negative control samples, or by PLIN1-APEX2 compared to combined negative control and Cyto-APEX2 (C,F,I), under basal (A-C), Fsk (D-F), or Fsk+Ins (G-I) treatment conditions.

**
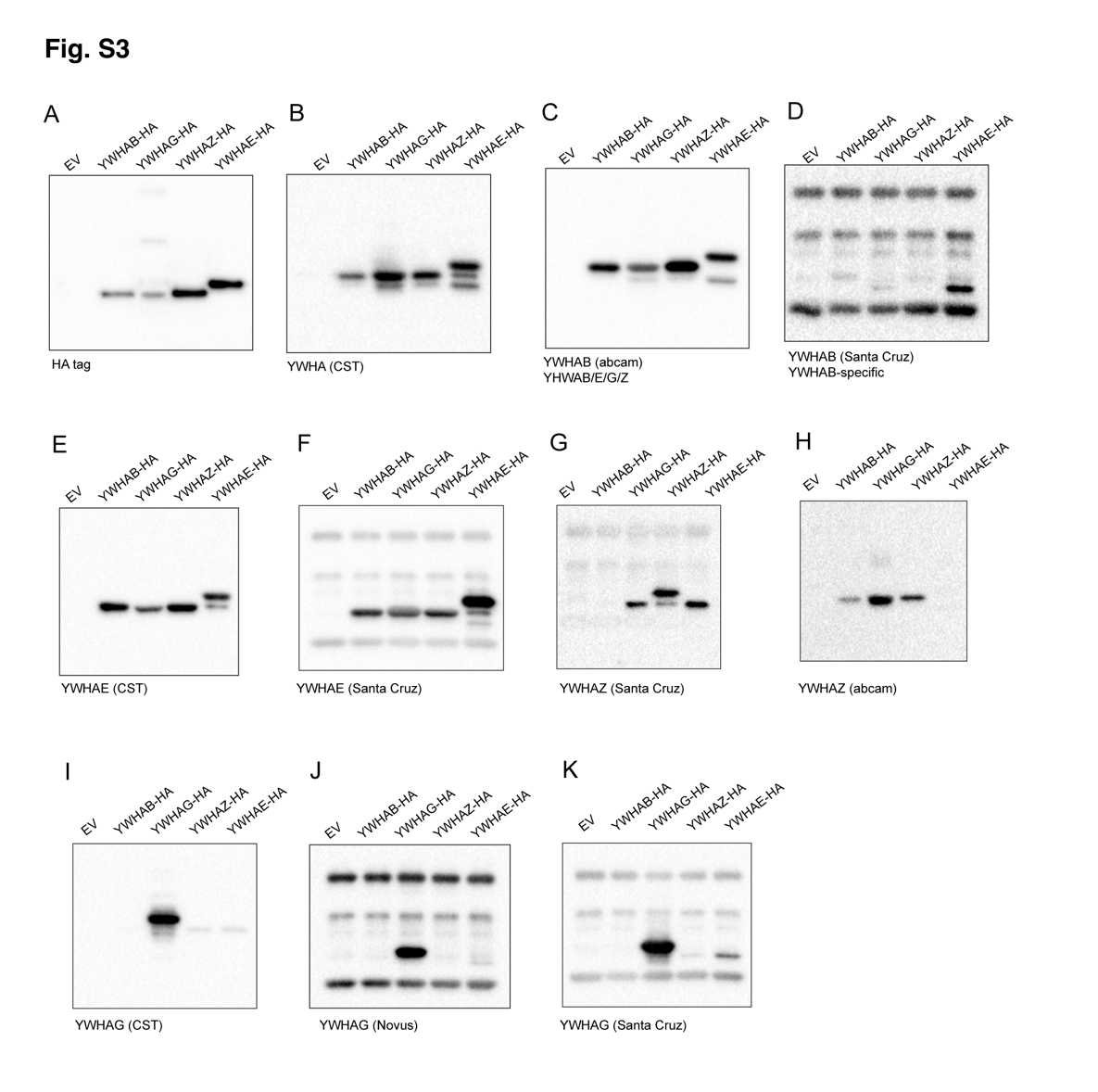
Figure S3. Variable specificity of commercial antibodies targeting 14-3-3 protein isoforms.** 293T cells were transfected by pcDNA3.1 empty vector or pcDNA3.1 vector containing YWHAB-HA, YWHAG-HA, YWHAZ-HA, or YWHAE-HA. Immunoprecipitated HA-tagged proteins were detected by western blotting using antibodies against: (A) HA tag , (B) YWHA pan from abcam , (C) YWHAB from abcam , (D) YWHAB from Santa Cruz, (E) YWHAE from CST, (F) YWHAE from Santa Cruz, (G) YWHAZ from abcam , (H) YWHAZ from Santa Cruz , (I) YWHAG from CST, (J) YWHAG from Novus , and (K) YWHAG from Santa Cruz. The heavy chain and light chains of mouse anti-HA antibody used for immunoprecipitation were also detected when primary antibodies to 14-3-3 isoforms were produced in mouse (D,F,G,J,K).
